# Phthalate Exposure: From Quantification to Risk Assessment

**DOI:** 10.3390/toxics10060330

**Published:** 2022-06-16

**Authors:** Lidia Caporossi, Maria Marino

**Affiliations:** 1 INAIL, Department of Occupational and Environmental Medicine, Epidemiology and Hygiene, Via Fontana Candida 1, 00078 Monteporzio Catone, Italy; 2 Department of Science, University of Rome Tre, Viale G. Marconi, 446, 00146 Rome, Italy

Phthalates (di-esters of phthalic acid) are a group of synthetic organic compounds present in the environment because of their wide use in a multitude of important industrial products (e.g., cosmetics, medical devices, plastics, and food packages), mainly as plasticizers to improve mechanical properties such as flexibility, transparency, durability, and longevity of polyvinyl chloride. The widespread occurrence of phthalates in the environment as well as their endocrine-disrupting and reproductive effects contribute to their recognition as substances of high concern [1,2,3,4]. In this Special Issue of *Toxics* entitled “Phthalate Exposure: From Quantification to Risk Assessment”, we collected both reviews and articles to identify the levels of these substances in the environment and in other sources of human exposure (e.g., occupational), their toxic effects, and their action mechanisms. Special attention was paid to analytical methods able to quantify low concentrations of phthalates in different complex matrixes, particularly in human biomonitoring.

Exposure to phthalates occurs through contaminated food ingestion; through skin absorption, i.e., in the case of cosmetic creams; or through inhalation [2]. A particular type of exposure is that which occurs by migration of phthalates from medical devices made of plastic, such as drip bags or tubes for therapies. The wide presence of these substances in living environments as well as in specific workplaces has allowed us to highlight their generalized exposure, with registered dosages in the general population, through surveys of biomonitoring in both the United States and Europe [5,6]. Women of childbearing age seem to have higher urinary phthalate levels than men, probably due to the use of personal care products [7]. The paper by Caporossi and colleagues [8] provided evidence of a relationship between the possibility of pregnancy loss, and exposure to diethylhexyl phthalate (DEHP) and diethyl phthalate (DEP). The presence of significantly higher values of certain phthalates, DEP metabolite in particular, especially in women with recurrent pregnancy loss and idiopathic infertility, suggests a possible involvement of these compounds as competing factors in reproductive issues [8]. These data require specific attention due to phthalate being widely use and to the absence of particular law restriction in Europe for DEP. Furthermore, studies carried out on male populations suggested that even for seminal fluid quality, phthalate exposure could be deleterious [9]. In particular, exposure to DEP seems to be linked with a reduction in semen concentration. Moreover, this type of exposure could also lead to increased oxidative stress, even in seminal fluid, because the production of oxidative stress is one of the possible mechanisms of endocrine dysfunction owing to chemicals, probably linked to an interference in antioxidant protective mechanisms [10,11]. Indeed, some researchers proposed reactive oxygen species (ROS) as possible biomarkers of toxicity [12]. The paper by Pigini and collaborators [13] documented the correlation between phthalates and ROS in two hundred and seventy-four couples undergoing an assisted reproduction technology treatment. In particular, 8-oxo-7,8-dihydro-2-deoxyguanosine (8-oxo-dGuo) was found to be associated with higher levels of DEHP, DnBP, and diiso-butylphthalate (DiBP) [13,14], and 8-oxo-7,8-guanosine (8-oxo-Guo) emerged as a really sensitive marker of oxidative stress in this type of exposure [15]. Of particular interest was the studies by Jenkins and colleagues carried out in the Neonatal Intensive Care Units [16], where the possibility of the exposure of an extremely fragile population to the pollutants released from medical devices was tested.

Zebrafish is a model widely applied in toxicology to estimated possible developmental risks for humans and for exposure to endocrine disrupter toxics [17,18]. The paper by Kwang and colleagues [19] collected data obtained from acute and chronic exposure of zebrafish to DEHP. This experimental model allowed them to study the toxic effects of phthalates starting from the embryonic stage to different developmental stages, thus obtaining a model that could facilitate risk assessments of DEHP exposure. Closely related to phthalate toxic effects is the paper by Liu and collaborators [20], who developed a 3D-QSAR model based on the investigation of the phthalates effects on nuclear factor erythroid 2 related factor 2 (NRF2), a nuclear transcription factor that regulates oxidative stress and plays a crucial role in liver detoxification. The experiments performed in human hepatocyte (HepG2) cells could help predict the toxicity of new analogues and provide a reference for the design of less toxic phthalates PAEs in the future.

The mechanisms at the root of the endocrine disruptor effects of phthalates has been addressed in the paper by Fiocchetti and colleagues [21]. These authors demonstrated that DEP could induce estrogen receptor α subtype (ERα) phosphorylation and degradation even without direct binding. In this way, DEP modulates estrogenic signaling through the indirect induction of changes in conformation in ERα and the corresponding functional outcomes and transduction mechanisms [21].

Finally, the validation and use of an analytical method useful for specific determination, for animals [22], for humans, and for studying the possible route of exposure are core questions for obtaining valuable findings. The analysis of phthalates represents a challenge for numerous researchers due to their, usually, low concentrations in different matrixes (food, human urine, different materials such as gloves [23], animal blood [22], and environmental matrixes). Furthermore, a specific complexity is linked to possible cross-contamination in the laboratory during the steps of quantification owing to plastic materials being used for laboratory analysis. During the years, for the pretreatment of samples, an evolution focused on quickness, simplicity, low sample handling, and the optimization of the use of solvents and reagents was documented. Both solid phase extraction (SPE) and liquid/liquid extract (LLE) were developed for the extraction of phthalates from different matrixes. Regarding analytical methods, the chromatographic techniques were the most commonly used, usually gas chromatography or high performance liquid chromatography (HPLC) coupled with mass spectrometry (MS) or triple quadrupole mass spectrometry (MS/MS) [23,24].

In Europe, the HBM4EU biomonitoring project was implemented [25] to understand background levels of phthalates in the general population due to daily environmental exposure. Urinary phthalate levels were analyzed in the recruited subjects. Within this project, reported in the paper by Mol and colleagues [26], the analytical, validation, and quality aspects of the data were crucial, and therefore, inter-laboratory evaluations were conducted to ensure the quality of the data produced.

In conclusion, the adverse effects of phthalates on animals and humans were highlighted in this Special Issue. In particular, the reprotoxicity of phthalates clearly emerged. Given the prevalence of infertility in the human population, estimated at about 15%, investigating any possible risk factors is significant. Furthermore, the industrial production of phthalates occurs, again, worldwide at very consistent levels; this call for further studies to promote healthy workplaces and to reduce possible environmental contamination. Because of the daily use of personal care products, the communication of potential health risks for consumers represents a specific target for future actions.
